# Patient Perspectives on Innovative Telemonitoring Enhanced Care Program for Chronic Heart Failure (ITEC-CHF): Usability Study

**DOI:** 10.2196/24611

**Published:** 2021-09-14

**Authors:** Sheau Huey Chen, Iain Edwards, Rajiv Jayasena, Hang Ding, Mohanraj Karunanithi, Alison Dowling, Jamie Layland, Andrew Maiorana

**Affiliations:** 1 School of Physiotherapy and Exercise Science Curtin University Perth Australia; 2 Department of Community Health Peninsula Health Melbourne Australia; 3 The Australian e-Health Research Centre Health and Biosecurity Unit Commonwealth Scientific & Industrial Research Organisation Melbourne Australia; 4 RECOVER Injury Research Centre Faculty of Health and Behavioural Sciences The University of Queensland Brisbane Australia; 5 The Australian e-Health Research Centre Health and Biosecurity Unit Commonwealth Scientific & Industrial Research Organisation Brisbane Australia; 6 PCH-Northside Clinical Unit School Faculty of Medicine The University of Queensland Brisbane Australia; 7 Department of Cardiology Peninsula Health Melbourne Australia; 8 Peninsula Clinical School Monash University Melbourne Australia; 9 Allied Health Department and Advanced Heart Failure and Cardiac Transplant Service Fiona Stanley Hospital Perth Australia

**Keywords:** chronic heart failure, telemonitoring, usability, acceptance, patient perspectives

## Abstract

**Background:**

Telemonitoring enables care providers to remotely support outpatients in self-managing chronic heart failure (CHF), but little is known about the usability and patients’ willingness to engage with this technology.

**Objective:**

This study aims to evaluate feedback from patients with CHF following participation in the Innovative Telemonitoring Enhanced Care program for CHF (ITEC-CHF) study.

**Methods:**

The telemonitoring intervention consisted of three components: remote weight monitoring, structured telephone support, and nurse-led collaborative care. Participants were provided with electronic weighing scales (W550; ForaCare), and a computer tablet (Galaxy Tab A; Samsung). They were asked to weigh themselves on the provided scales daily. Telemonitoring was integrated with a personal assistance call service and a nurse care service according to their workflows in usual care. Feedback on the usability of ITEC-CHF was collected via survey from study participants following 6 months of receiving telemonitoring care for their body weight. Survey responses were provided on a 5-point Likert scale and through open-ended questions to determine participants’ perceived benefits and barriers to using ITEC-CHF.

**Results:**

A total of 67 participants (49/67, 73% male), with a mean age of 69.8 (SD 12.4) years completed the survey. The majority of participants agreed or strongly agreed that the ITEC-CHF program was easy to use (61/67, 91%), easy to navigate (51/65, 78%), useful (59/65, 91%), and made them feel more confident in managing their weight (57/67, 85%). Themes related to participants’ perceptions of telemonitoring included increased support for early intervention of clinical deterioration, improved compliance to daily weighing, a sense of reassurance, and improved self-care and accountability, among others.

**Conclusions:**

ITEC-CHF was rated highly on usability and was well accepted by users as part of their routine self-management activities. Participants were willing to use telemonitoring because they perceived a broad spectrum of benefits for CHF management.

**Trial Registration:**

Australian New Zealand Clinical Trial Registry ID ACTRN 12614000916640; https://www.anzctr.org.au/Trial/Registration/TrialReview.aspx?id=366691.

## Introduction

Chronic heart failure (CHF) is a complex disease that is expensive to manage and affects approximately 2%-3% of the adult population [1], with a prevalence that continues to increase [2]. Daily weight monitoring and symptom control are cornerstones of CHF management [3]; hence, innovative strategies that are both effective and acceptable to patients are required to support traditional approaches to manage these aspects of care. Recent studies have reported that remote monitoring can improve health outcomes and reduce costs associated with CHF care by providing real-time physiological information to health care providers that can be acted on quickly, reducing the potential for progressive clinical deterioration and more complex care requirements [4,5]. These contemporary telemonitoring systems have the advantage of being delivered by portable devices, enabling patients to be monitored in real time from anywhere with access to the internet [6]. However, positive findings of the efficacy of telemonitoring in CHF management are not ubiquitous, with several studies identifying patients who are resistant to change [7-9].

The mixed results from telemonitoring studies may, in part, reflect the willingness or readiness of patients with CHF to engage with telemonitoring technology and to adhere to its use [10-12]. Because the prevalence of CHF increases with age, a high proportion of patients with CHF are over 75 years of age. This is a subset of the population in whom digital literacy has historically been low. However, the characteristics of the “over 75 years” demographic in modern times is different than that in prior generations, with increased life expectancy [13] and rapidly improving digital literacy [12] highlighting the need for new research in this area.

Although several recent studies have investigated the perceptions of telemonitoring in other clinical cohorts, such as patients with chronic kidney disease, chronic obstructive pulmonary disease, or hypertension [10,11,14,15], there are few contemporary studies describing the perceptions of telemonitoring in patients with CHF. Remote monitoring in patients with CHF has specific objectives and unique challenges. For example, rapid fluctuations in body weight (>2 kg in 48 hours) may be the result of a variety of precipitating factors such as poor adherence to fluid and salt restrictions or noncompliance with medication, which can be rectified through modification of self-care behaviors, or it may be attributed to cardiac deterioration warranting urgent medical support [16]. This increases the complexity of telemonitoring and emphasizes the importance of integrated clinical support in telemonitoring ecosystems [16,17], highlighting the importance of user-friendly technology [18-20].

The Innovative Telemonitoring Enhanced Care program for CHF (ITEC-CHF) was the first such program to incorporate telemonitoring supported by a 24-hour call center and first-line nurse-led CHF intervention in community care settings in Australia [21,22]. To minimize weight monitoring burdens and technical difficulties, the program introduced a novel “zero-touch” design, meaning that the participants were not required to interact with the technology other than stepping onto a scale for weight measurement as in usual care, and they did not need to have extra knowledge or skills to receive the telemonitoring intervention [21,22]. The objective of this study was to assess perceptions of telemonitoring among patients with CHF who participated in the ITEC-CHF study and to evaluate the usability of this model of care.

**Figure 1:**
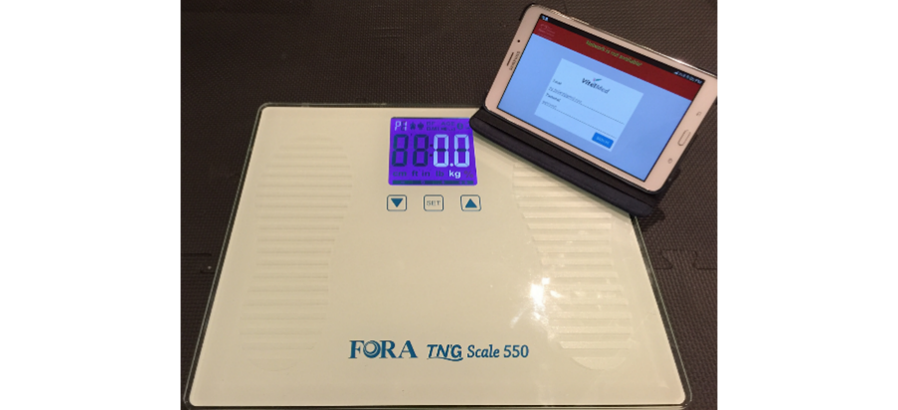
ITEC-CHF Telemonitoring System. ITEC-CHF: Innovative Telemonitoring Enhanced Care Programme for Chronic Heart Failure.

## Methods

### Study Setting and Design

A detailed description of the protocol for the ITEC-CHF study has been previously published [23]. Participants were recruited from the Frankston Hospital and Rosebud Hospital in Victoria, Australia, and Royal Perth Hospital and Fiona Stanley Hospital in Western Australia. Between January 2016 and December 2017, a total of 91 participants enrolled in the ITEC-CHF trial were mailed a survey and provided with a self-addressed and stamped envelope to return the survey at the end of the 6-month intervention. The survey consisted of two parts (see Multimedia Appendix 1). Part 1 was designed to evaluate the usability of the ITEC-CHF telemonitoring system and consisted of 9 questions, which were scored on a 5-point Likert scale (1=strongly disagree; 2=disagree; 3=neither agree nor disagree; 4=agree; and 5=strongly agree). The 9 Likert scale questions addressed the following concepts adapted from the technology acceptance model (TAM) and attitude toward technology use: (1) ease of use, (2) participants’ confidence with managing CHF, (3) participants’ ability to navigate the technology, and (4) perceived usefulness [23-26]. These questions assessed the participants’ perceptions of telemonitoring and their comfort with using the technology involved. The TAM is an information technology framework for understanding users’ adoption and use of emerging health care technologies [25,26]. The model states that usefulness and ease of use are two essential elements in describing participants’ attitudes when using a new technology [26]. A number of studies support the validity of the TAM and its satisfactory explanation of end-user system usage [23,24,27]. Part 2 of the survey involved 3 open-ended questions to provide the participants an opportunity to express more detailed opinions about the ITEC-CHF telemonitoring system. The open-ended questions addressed perceived benefits and perceived barriers, as well as sought participants’ suggestions about improving the system. The estimated time to complete all questions was approximately 15 minutes.

### ITEC-CHF Telemonitoring System

Eligible participants for the survey were required to have completed the ITEC-CHF intervention. The detailed protocol for this study has been published [22], but it is summarized as follows:

Participants were provided with electronic weighing scales (W550; ForaCare), and a computer tablet (Galaxy Tab A; Samsung). They were asked to weigh themselves on the provided scales daily. The measured weight entry was recorded in the weighing scale and then automatically transmitted to the tablet via a wireless Bluetooth function embedded in the scales. The tablet was preloaded with an Android application (MedTech Global) that received the weight entry and uploaded it to a proprietary software package, ManageMyHealth (MedTech Global). A web application in MMH automatically monitored the uploaded weight entries in real time to generate alerts and triage those alerts to project nurses and the call center. The alerts were designed in accordance with the National Heart Foundation of Australia’s *Guidelines for the Prevention, Detection, and Management of Chronic Heart* [17].

The telemonitoring intervention consisted of three components: remote weight monitoring, structured telephone support, and nurse-led collaborative care. Telemonitoring was integrated with a personal assistance call service (MePACS) and a nurse care service according to their workflows in usual care.

Operators at the call center responded to the alerts in real time (24 hours, 7 days a week). In cases where the participant required clinical support, such as advice for assessing CHF symptoms or managing fluid and salt restriction, the call operator arranged a nurse follow-up.

The project nurses provided structured interventions according to three types of alerts: rapid weight fluctuation (±2 kg in 2 days), slow weight fluctuation (±5 kg in 28 days), and low-risk weight fluctuation (±1 kg over 24 hours). If a participant’s body weight fluctuation exceeded ±1 kg (but less than ±2 kg) over 24 hours, a questionnaire was automatically triggered and sent to the participant’s computer tablet. If the participant reported any of the clinical conditions in the questionnaire or did not respond to the questionnaire, the project nurses contacted the participant for a clinical assessment. However, if the response to the questionnaire determined the participant was asymptomatic, the alert was cancelled automatically to minimize unnecessary alerts to the project nurses.

### Inclusion and Exclusion Criteria

The study’s inclusion criteria were as follows: patients (1) with CHF diagnosed by a clinician with an ejection fraction ≤40%, (2) who were able to weigh oneself safely, (3) who were at least 18 years of age, (4) who have a regular personal general practitioner (GP) or agree to use a designated GP, (5) who have a permanent residential address, and (6) without significant cognitive impairment. The exclusion criteria were as follows: (1) patients with expected survival <12 months, (2) patients with end-stage renal failure on dialysis, (3) long-term nursing home residents, or (4) patients participating in any other clinical trial. All participants provided written informed consent.

### Statistical Methods

Statistical analyses were performed using SPSS software (version 26.0; SPSS Inc.). Descriptive statistics (mean and SD, frequencies, and percentages) were used to characterize the study population and described participants’ perceptions of usability of ITEC-CHF.

Open-ended questions were transcribed and imported into NVivo version 12 (QSR International) to facilitate the coding and to maximize the effectiveness and efficiency in sorting and merging the data according to themes reflecting common views and experiences. These were collated and supported by deidentified quotes from participants. Thematic analysis was performed to identify themes related to participants’ perceptions of the perceived benefits and perceived barriers, as well as their suggestions about improving the system, thus capturing participants’ understandings and allowing an in-depth analysis of the data [24]. Data were described, summarized, and then interpreted in relation to broader implications. The first author (SC), who is a nurse researcher with experience of research on CHF telemonitoring, familiarized herself with the data by reading the participants’ responses several times, while taking notes. Points of interest were noted while reading and re-reading the transcripts. Following production of an initial set of codes, a thematic map was developed, which presented themes and subthemes. Accounts were then re-read to ensure that coding was checked and that nothing had been overlooked. Themes and subthemes were then allocated. The last author (AM), who is an experienced researcher in the fields of cardiac rehabilitation and heart failure management, cross-checked the set of themes and was fully involved in the data interpretation and write-up for dissemination.

### Ethics Approval

The ethics application for the trial site in Victoria has been approved by Peninsula Health Human Research Ethics Committee (HREC Reference: HREC/14/PH/27), and the ethics applications for trial sites in Western Australia have been approved by Royal Perth Hospital Human Research Ethics Committee (Reference: 15-081) and the Curtin University Human Research Ethics Committee (Reference: HR 181/2014). This study complies with the Declaration of Helsinki. The trial has been registered in the Australian New Zealand Clinical Trials Registry, Trial ID: ACTRN12614000916640.

## Results

### Overview

The survey response rate was 77% (67/91 surveys; Table 1). There were no significant differences between the demographics or clinical characteristics of the participants who completed the survey and the overall cohort who completed the ITEC-CHF study.

For the broad concepts of *ease of use*, *confidence*, *navigability,* and *usefulness* described in the TAM, 91% (61/67) of participants “agreed” or “strongly agreed” that the telemonitoring system was easy to use, 85% (57/67) “agreed” or “strongly agreed” that the technology improved their confidence in managing their CHF condition, 78% (51/65) “agreed” or “strongly agreed” that the technology was easy to navigate, and 91% (59/65) “agreed” or “strongly agreed” that the telemonitoring was useful. A few participants indicated that they “disagreed” or “strongly disagreed that the telemonitoring system was easy to use (3%), that the technology improved their confidence in managing their CHF condition (2%), that the technology was easy to navigate (2%), and that the telemonitoring was useful (2%).

Table 2 presents the information related to the 9 questions that were rated on a 5-point Likert scale. Each indicator was evaluated across multiple questions.

**Table 1:** Demographics and clinical characteristics of study participants.

Characteristic		Value		*P* value
		Completed survey (n=67)	Completed ITEC-CHF^a^ (N=91)	
Age in years, mean (SD)		69.8 (12.4)	69.5 (12.3)	.89
**Gender, n (%)**				
	Male	49 (73)	66 (73)	.94
	Female	18 (27)	25 (27)	.95
**Highest education achieved, n (%)**				
	Less than high school	9 (13)	10 (11)	.64
	High school	28 (42)	41 (45)	.68
	Trade or technical training	8 (12)	12 (13)	.81
	College or university undergraduate	19 (28)	23 (25)	.67
	Postgraduate	3 (4)	5 (5)	.78
BMI, mean (SD)		32.1 (10.6)	31.4 (9.6)	.86
**NYHA^b^, n (%)**				
	I	5 (7)	8 (9)	.77
	II	50 (75)	68 (75)	.99
	III	11 (16)	14 (15)	.86
	IV	1 (1)	1 (1)	.83
LVEF^c^ (%), mean (SD)		28.7 (7.7)	29.1 (7.1)	.97
**Other medical conditions, n (%)**				
	CHD^d^	46 (68)	58 (64)	.52
	COPD^e^ or asthma	16 (24)	23 (25)	.74
	CKD^f^	7 (10)	10 (11)	.75
	T2DM^g^	22 (33)	28 (31)	.87

^a^ITEC-CHF: Innovative Telemonitoring Enhanced Care Programme for Chronic Heart Failure

^b^NYHA: New York Heart Association Functional Classifcation.

^c^LVEF: left ventricular ejection fraction.

^d^CHD: coronary heart disease.

^e^COPD: chronic obstructive pulmonary disease.

^f^CKD: chronic kidney disease.

^g^T2DM: type 2 diabetes mellitus

**Table 2:** Respondents’ grading based on usability survey questions.

		Value, n (%)						Score, mean (SD)
Survey item		Strongly disagree	Disagree	Neither agree nor disagree	Agree	Strongly agree		
**Ease of use (n=67)**								
	The weighing scale was easy to use	1 (1.5)	1 (1.5)	3 (4.5)	9 (13.4)	53 (79.1)	4.7 (0.8)	
	The touch screen tablet was easy to use	0 (0)	3 (4.5)	8 (11.9)	17 (25.3)	39 (58.2)	4.4 (0.9)	
	The information given to me in how to weigh myself using the device was easy to understand	0 (0)	0 (0)	2 (3)	25 (37.3)	40 (59.7)	4.6 (0.6)	
**Confidence (n=67)**								
	The technology helped me to manage my chronic heart condition	0 (0)	2 (3)	2 (3)	33 (49.3)	30 (34.3)	4.1 (0.8)	
	I feel more confident about managing my chronic heart failure after taking part in this research project	0 (0)	0 (0)	2 (3)	34 (50.8)	13 (35.8)	4.2 (0.7)	
**Navigability**								
	I found the weight reminders helpful on the touch screen tablet (n=56)	0 (0)	0 (0)	11 (19.6)	22 (39.3)	23 (41.1)	4.2 (0.8)	
	I found the symptom questions easy to respond to on the touch screen tablet (n=65)	0 (0)	3 (4.6)	13 (20)	20 (30.8)	29 (44.6)	4.2 (0.9)	
**Usefulness**								
	When I forgot to weigh myself, I found the reminder calls helpful (n=63)	1 (1.6)	1 (1.6)	6 (9.5)	17 (27)	38 (60.3)	4.4 (0.9)	
	When my weight changed, I found the call from the Chronic Heart Failure nurse helpful (n=65)	1 (1.5)	0 (0)	3 (4.6)	23 (35.4)	38 (58.5)	4.5 (0.7)	

### Themes and Subthemes Analyzed

Participants provided feedback, including a range of benefits and barriers to using telemonitoring. Eight key themes related to the ITEC-CHF program emerged from responses to the open-ended questions. Quotes from participants are provided to support each theme.

#### Increased Support for Early Intervention of Clinical Deterioration

Clinicians were able to view patient health data easily and quickly, which enabled early detection of clinical deterioration. This meant that problems were detected quickly, and participants were able to receive an early intervention.

Weight fluctuation detected early and see GP same day.

#### Improved Compliance to Daily Weighing

The telemonitoring system helped participants get into a routine and inform them when a change occurred in their weight that was outside the predetermined limits.

Information exchange. Motivation to try and be healthy.

Learning about weight changes and fluid balance.

#### A Sense of Reassurance

Participants indicated they felt reassured that a clinician was behind the scenes reviewing their data.

Staff are competent.

Safety net that someone is watching.

#### Improved Self-care and Accountability

Participants felt accountable for their self-management because they were being monitored and would receive a reminder if they missed weighing themselves. This was reported as having had a positive effect on compliance to their self-management regime.

Weight measurement helped me with trying to maintain my health status.

Made me personally more accountable of fluid management.

Encouraging to weigh regularly. Help keep an eye on my diet.

#### Supportive of Self-Management

The ITEC-CHF environment helped participants feel supported in self-managing their condition while reflecting on the telemonitoring system in self-care.

Weighing reminders from MEPACS.

Don't feel alone. Familiar with nurses.

Reassuring that help is on hand.

#### Technical Difficulties

Some concerns expressed by participants were related to the technology, mainly due to Bluetooth connectivity issues in the early stages of the trial.

When machine doesn't register (scales).

Computer tablet not registering weight measured from scales.

#### Flexibility of Telemonitoring System

Some participants suggested they would have liked greater flexibility to be able to weigh themselves later than 10 AM to suit their lifestyle. This feedback was provided by participants who are employed, including those who work a night shift, to have the flexibility of the cutoff time to weigh in extended.

Sunday mornings when woken by MEPACS.

Extend time to midday.

Extend time limit.

#### System Not Suitable for All Patients

Participants who had lifestyles involving frequent traveling found the continuous telemonitoring unsuitable. In addition, some participants reported difficulty in answering the questions on the computer tablet in a timely manner.

Not suitable when going away on holiday.

Not enough time to answer symptoms questions.

## Discussion

### Principal Findings

In this evaluation of the perceptions of telemonitoring among patients with CHF, the majority of participants “agreed” or “strongly agreed” that the intervention was feasible and helpful in their care. This included being easy to use (91% agreement) and helpful in improving their confidence in self-management (85% agreement). These findings are consistent with those reported from studies in other cohorts of people with chronic diseases that have evaluated perceptions of telemonitoring [10,11,14,15], but these results also provide new insights into the perceptions of patients with CHF.

Feedback from participants in this study highlights the importance of minimal user burden and ensuring user-friendly technology for telemonitoring to be acceptable to patients. High rates of satisfaction were observed with all the aspects of usability surveyed. Participants reported that the ITEC-CHF program was easy to use, easy to navigate, useful, and increased their confidence in managing their weight. Similarly, patients with chronic kidney disease were found to be highly accepting of using telemonitoring because they perceived it as being interactive and applicable in managing their condition [10]. In patients with hypertension, high levels of acceptability in using telemonitoring that relates to user-friendly technology has been previously reported [11]. User acceptance is especially important if telemonitoring is to be widely adopted; this is an important objective in the COVID-19 era when remotely delivered health care is increasingly being utilized to avoid subjecting patients to the risk of infection.

Compliance with care provider instructions and being self-disciplined in health management activities and self-care were two themes that were expressed by a high proportion of participants using the ITEC-CHF system. Compliance with self-care activities, such as diet, exercise, and medication adherence, are important factors in managing chronic conditions such as CHF given that successful disease management is, in part, dependent on patients’ ability and willingness to carry out self-care activities [17]. Moreover, confidence in undertaking self-management activities, particularly the ability to reliably self-identify symptoms associated with clinical deterioration and take appropriate action in a timely manner is an important component of chronic disease management [28]. The observation that telemonitoring is beneficial for weight surveillance represents an important clinical outcome given that fluctuations in body weight are a reliable way of detecting fluid imbalance, which can be associated with poor self-care compliance or disease exacerbation [29-31].

However, the acceptance of telemonitoring was not ubiquitous for participants in the current study. For example, the technology in its current form may not suit patients who travel frequently. Several participants also indicated that greater flexibility in the telemonitoring system would reduce disruption to their lives, especially during holidays and on weekends. It was suggested by some participants that having the ability to alter the time before an alert was sent (ie, changing it to after 10 AM) would reduce the psychological burden of the alert system during these periods. This is an important consideration because previous studies have found that insufficient flexibility in telemonitoring models may hinder the ongoing use of the system [32-36].

Participant feedback also highlighted the importance of engaging consumers with a lived experience of CHF in the co-design of telemonitoring to ensure that it is simple and easy to engage with by the end user. Participants stressed the importance of a system that is robust, with easily accessible technical support—a finding consistent with observations in other clinical groups [37,38]. This is critical because technical problems are known to be a significant impediment to the uptake and adherence to telemonitoring [30-32]. From the ITEC-CHF trial, it was evident that technical issues led to disengagement from the system when encountered by some participants [22]. Patients with CHF often have multiple competing comorbid health issues to manage in their lives, so a seamless system of telemonitoring takes on additional importance.

### Limitations

There are several limitations to the study that warrant highlighting. First, the results from the usability of the ITEC-CHF program were based on a relatively small sample size, so larger studies are required to confirm these findings. Second, there was no baseline data of participants’ perceptions of the usability of the system to provide a comparison for user satisfaction measured at the end of the study. However, this design would have its own limitations because participants would lack the experiential insight derived from being involved in the trial to answer some of the questions at baseline. Third, the findings are based on the experiences of participants who completed the trial and who are, therefore, likely to have a more favorable view of the telemonitoring system than those who dropped out. Finally, the single-group ITEC-CHF usability design precluded the assessment of the feasibility of randomization procedures, attrition, outcome measures, and acceptability in a control arm.

### Conclusions

In this study evaluating the usability of a telemonitoring program in patients with CHF, a high overall usability rating was achieved, and the telemonitoring system was generally well accepted by users as an adjunct to their routine self-management activities. Participants in the study expressed that they were confident in using the ITEC-CHF system and reported many perceived benefits, including quick identification of early signs of clinical deterioration, which allows for faster response to manage the symptoms of CHF. Future trials that are powered to assess whether telemonitoring effects rehospitalization and mortality rates are required to determine whether these characteristics of telemonitoring translate to an improvement in clinical outcomes for patients with CHF.

## Appendix

Multimedia Appendix 1 Innovative Telemonitoring Enhanced Care Program for Chronic Heart Failure (ITEC-CHF) participant evaluation form.

## Abbreviations

CHF: chronic heart failure
HREC: Human Research Ethics Committee
ITEC-CHF: Innovative Telemonitoring Enhanced Care program for CHF
TAM: technology acceptance model
