# Supplementary material for: Patient Perspectives on Innovative Telemonitoring Enhanced Care Program for Chronic Heart Failure (ITEC-CHF): Usability Study
Source: JMIR Cardio. 2021 Sep 14;5(2):e24611. doi: 10.2196/24611 (PMC8479597; doi:10.2196/24611)
Supplement: Multimedia Appendix 1 [file cardio_v5i2e24611_app1.docx]

Appendix 1

**The Innovative Telemonitoring Enhanced Care program**

**for CHF (ITEC-CHF)**

**Participant Evaluation Form**

Thank you for participating in the Chronic Heart Failure Study.

The study team would appreciate your feedback on your experiences as a participant of the Chronic Heart Failure Study.  This will help us identify what worked well and what did not work so well.  This information will be very helpful for our planning of future research studies.

Once you have completed the questions, please return the form to the research nurse.

Please indicate your rating of the following statements by circling the appropriate number, using the following scale:-

1=strongly disagree

2=disagree

3=neither agree nor disagree

4=agree

5=strongly agree

|  | **Statement** | **Strongly disagree** | **Disagree** | **Neither agree or disagree** | **Agree** | **Strongly agree** |
| --- | --- | --- | --- | --- | --- | --- |
| 1 | The weighing scale was easy to use | 1 | 2 | 3 | 4 | 5 |
| 2 | The touch screen tablet was easy to use | 1 | 2 | 3 | 4 | 5 |
| 3 | The technology helped me to manage my chronic heart condition | 1 | 2 | 3 | 4 | 5 |
| 4 | I feel more confident about managing my chronic heart failure after taking part in this research project | 1 | 2 | 3 | 4 | 5 |
| 5 | I found the weight reminders helpful on the touch screen tablet | 1 | 2 | 3 | 4 | 5 |
| 6 | I found the medication reminders helpful on the touch screen tablet | 1 | 2 | 3 | 4 | 5 |
| 7 | I found the symptom questions easy to respond to on the touch screen tablet | 1 | 2 | 3 | 4 | 5 |
| 8 | When I forgot to weigh myself, I found the reminder calls from MEPACS helpful | 1 | 2 | 3 | 4 | 5 |
| 9 | When my weight changed, I found the call from the Chronic Heart Failure nurse helpful | 1 | 2 | 3 | 4 | 5 |
| 10 | The information given to me in how to weigh myself using the device was easy to understand | 1 | 2 | 3 | 4 | 5 |

1. What did you find most beneficial about participating in the study?

……………………………………………………………………………………………………………

……………………………………………………………………………………………………………

2. What did you find the most difficult about taking part in the study?

……………………………………………………………………………………………………………

……………………………………………………………………………………………………………

3. Do you have any suggestions on how we can improve the study to make it more helpful?

……………………………………………………………………………………………………………

……………………………………………………………………………………………………………
